# Posttraumatic True Aneurysm of the Axillary Artery Following Blunt Trauma

**DOI:** 10.1155/2010/210391

**Published:** 2010-08-25

**Authors:** Tugrul Goncu, Faruk Toktas, Osman Tiryakioglu, Gunduz Yumun, Sinan Demirtas, Senol Yavuz

**Affiliations:** Department of Cardiovascular Surgery, Bursa Yuksek Ihtisas Education and Research Hospital, 16320 Bursa, Turkey

## Abstract

The majority of the axillary artery aneurysm cases arise as pseudoaneurysms secondary to blunt or iatrogenic trauma. Isolated traumatic true axillary artery aneurysm is a relatively unusual disorder and generally occurs with repetitive blunt trauma. A 22-year-old female patient with distal axillary artery true aneurysm due to simple blunt axillothoracic trauma is presented. The aneurysm was excised with subpectoral-axillary approach and saphenous vein graft interposition was applied. Long-term follow-up with the patient was uneventful.

## 1. Introduction

An aneurysm is defined as a permanent localized dilatation of an artery having at least a 50% increase in diameter compared with the expected normal diameter [1]. It may involve the entire wall of the vessel (true aneurysm) or only a portion of the wall and surrounding tissue (pseudoaneurysm) [1]. Pseudoaneurysm is characterized by focal defect in the arterial wall, with hemorrhage controlled by surrounding tissues [1]. This type constitutes the majority of the traumatic axillary aneurysms [2]. Traumatic true axillary artery aneurysms are relatively rare and generally occur with repetitive blunt trauma [3]. In this paper, we present an illustrative case with true axillary artery aneurysm following simple blunt axillo-thoracic trauma.

## 2. Case

A 22-year-old female was admitted our hospital with a gradually enlarging right axillary pulsatile mass. She complained of right upper extremity weakness, and coldness and pain after exercise. She had a history of right axillo-thoracic blunt trauma three months ago. She had noticed the mass 2 months before, and it was gradually enlarging since that time. 

**Figure 1:**
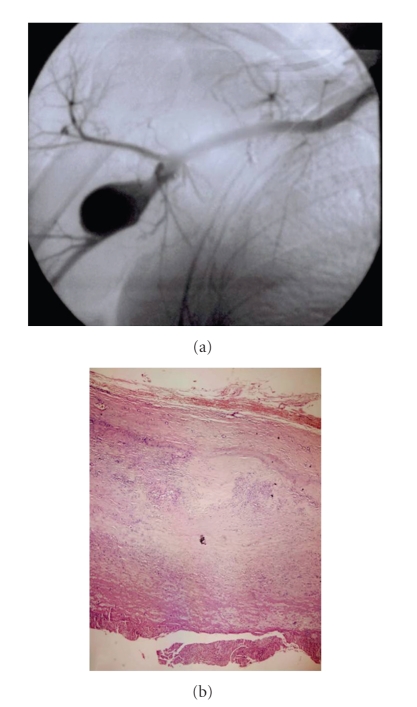
Digital subtraction angiography confirmed an aneurysm at the distal part of right axillary artery (a). In the presented case, pathologic examination of aneurismal sac revealed that all three layers of the arterial wall were intact despite various degrees of degeneration and fibrosis in the media layer (b). H&Ex100.

In our physical examination we found an about 3 × 4 cm solid, palpable pulsating mass in the right axillary region. Brachial blood pressures were 90/50 mmHg on right side and 110/75 mmHg on left side. Upper extremity arterial pulses in right side were poor in comparison with the left side. On auscultation a systolic murmur which was spreading to right axillary region had been heard on the mass. Neurological examination showed a mild loss of sensation in the left arm. There were no signs of vasculitis or connective tissue diseases associated with arterial involvement such as hyperelastic skin, hypermobile joints, or marfanoid habitus. Laboratory examinations, including erythrocyte sedimentation rate, C reactive protein, complete bloodcount, serological test for syphilis, rheumatoid factor, antinuclear antibody, antithrombin III, protein C, and protein S, were normal except for hypercholesterolaemia. A color Doppler ultrasound suggested the presence of an axillary artery aneurysm. Digital subtraction angiography confirmed a 4 × 6 cm fusiform aneurysm at the distal part of right axillary artery (Figure 1). Thoracic outlet syndrome was excluded by means of careful clinical, radiological, and electrophysiological examinations. 

**Figure 2:**
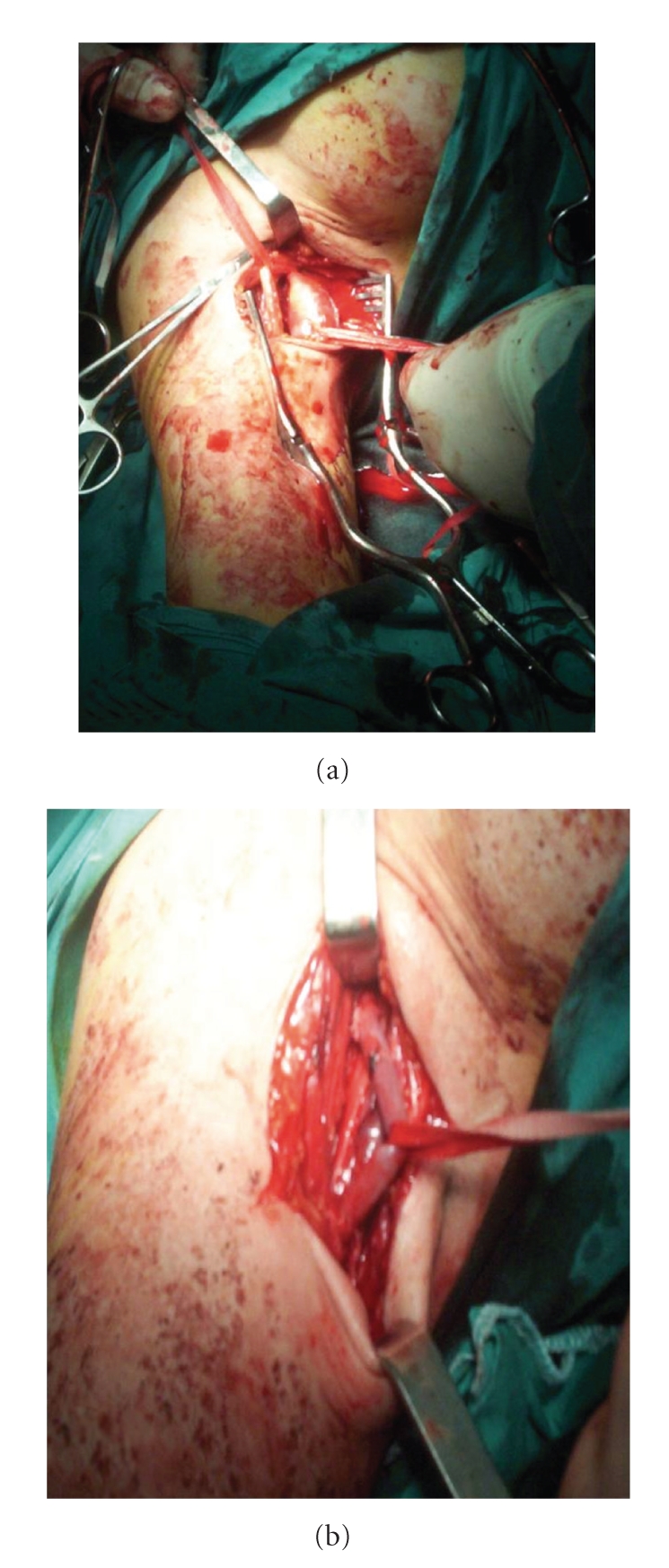
The aneurismal segment was resected and a saphenous vein graft was interposed between the two transected segments of axillary artery.

Under general anesthesia, we exposed the aneurysm by using the subpectoral-axillary approach. We encountered a large amount of organized thrombus when entering the aneurysm sac. An embolectomy was done using a Fogarty catheter but no embolism was detected. The aneurysm was excised. An adequate calibrate and length saphenous vein graft was interposed between the two transected segment of the axillary artery (Figures 2(a) and 2(b)). After the operation all of the right upper extremity peripheral pulses were pulsatile and both upper extremities blood pressures were approximately equal (115/75 mmHg and 110/75 mmHg resp.) at brachial level. The postoperative course was uneventful and the patient was discharged on the fifth postoperative day. She was symptom-free at follow-up three years later. 

Histopathological study of the specimen obtained from the aneurysmal sac revealed that all three layers of the arterial wall were intact. Degeneration and fibrosis were seen in the media layer (Figure 1(b)). 

## 3. Discussion

Pseudoaneurysms represent the majority of reported traumatic axillary artery aneurysms [2]. The majority of cases are due to penetrating or blunt trauma and less frequently due to congenital arterial defects, infection, and periarteritis nodosa. They may also be due to postobstructive lesions in patients with thoracic outlet syndrome [4]. Repeated blunt trauma to the axillary artery, classically due to long-term crutch use, may result in the development of an aneurysm. Thus, it is believed that blunt repetitive trauma weakens the arterial wall. False aneurysms of the axillary artery usually occur with penetrating trauma but may also occur with blunt trauma in the form of humeral fractures and anterior dislocation of the shoulder [3]. In the latter instance, the mechanism may be avulsion of tethered thoracoacromial, subscapular, or circumflex humoral vessels at the time of dislocation [3]. 

In the presented case, pathologic examination of aneurismal sac revealed that all three layers of the arterial wall were intact despite of in various degrees of degeneration and fibrosis in the media layer. These findings proved it to be a true aneurysm. We propose that compression of the arterial wall secondary to trauma produces a contusion of the arterial media with subsequent weakening of the wall and fusiform dilatation. This process contrasts the formation of pseudoaneurysms, which result when fibrous tissue surrounds a posttraumatic hematoma that is continuous with arterial flow. Although our findings, obtained through patient anamnesis, physical examination, and laboratory analysis, pointed away from the possibility that this aneurysm might have been present before the trauma, it is impossible to completely exclude this possibility.

Blunt trauma is often more difficult to diagnose and manage, and it can be easily overlooked, because the superficial evidence of injury is often absent even with an underlying vascular injury [5]. Difficulty in diagnosing the lesion clinically arises from the particular location of such lesions and the richness of the brachial and axillary artery collaterals [3, 5]. This latter fact explains why an ischemic syndrome is rarely observed. 

The recognition and treatment of these aneurysms are important because they can cause major disability, and lead to limb and digit loss. In addition to rupture, proximal aneurysms are complicated by thromboembolism with signs of an ischemic upper extremity, and symptoms, including gangrene, neuromuscular and sensory dysfunction from brachial plexus compression [3, 4]. In the presented case, neurological examination showed a mild loss of sensation in the left arm. The lack of neurological deficits immediately after injury and emerging gradually over time suggested that the loss of sensation might be due to compression of the aneurysm on brachial plexus. Arteriography is the essential diagnostic method, and magnetic resonance angiography and duplex scanning can be helpful for planning the operation [3, 6].

Elective surgical treatment has little risk to the patient and prevents the need for emergency operations [2–4]. Subpectoral-axillary exposure is fairly simple, requiring little dissection, being rather atraumatic, and having few disadvantages [7]. Prosthetic reconstruction of the axillary artery has been successful; however, because of the superior patency of vein grafts in upper extremity reconstructions, these are preferred [8]. Endovascular stent grafts have also been successfully used to treat these aneurysms [2–4]. 

## 4. Conclusion

True isolated axillary artery aneurysms due to blunt trauma are rare, but potentially dangerous lesions that threaten the upper extremity with vascular and neurologic compromise. They also have to be differentiated from false aneurysms. The occurrence of axillary artery injury and aneurysm formation after blunt trauma without shoulder dislocation and bone fracture is very rare, but is possible, as seen in this case. Most can be treated effectively with surgical excision and vascular grafting.
